# Supplementary material for: In Vitro Analysis of SARS-CoV-2 Spike Protein and Ivermectin Interaction
Source: Int J Mol Sci. 2023 Nov 16;24(22):16392. doi: 10.3390/ijms242216392 (PMC10671224; doi:10.3390/ijms242216392)
Supplement: Supplementary file 1 [file ijms-24-16392-s001.zip › ijms-2601295-supplementary.pdf]

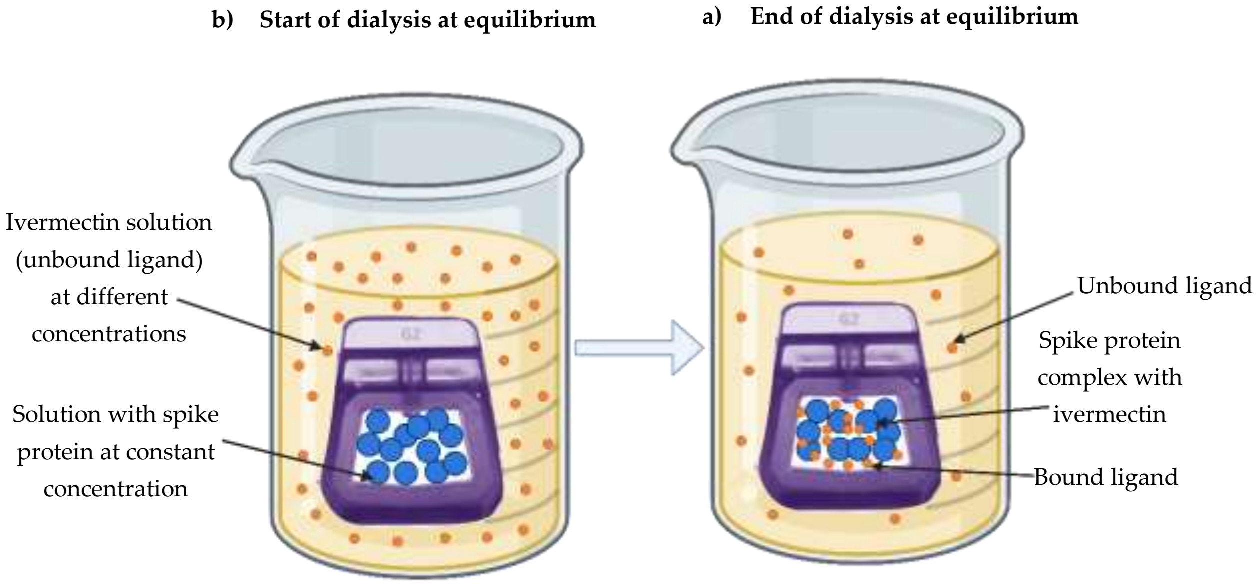

Figure S1. Equilibrium dialysis technique.

Table S1. Ligand bound and unbound ivermectin at different concentrations with recombinant S1 protein.

Reciprocal absorbance Ligand bound 1/ (A-A0)	Reciprocal[1] Ligand bound (μM) 1/ (bound)	Reciprocal[1] Ligand unbound (μM) 1/ (unbound)
1.64	0.0195	0.0615
1.58	0.0188	0.0666
2.03	0.0237	0.2971
3.05	0.0345	0.2482
6.49	0.0661	0.4602
12.87	0.1104	1

[1]Values obtained for the reciprocals of the bound and unbound ligand in the recombinant S1 protein with ivermectin using the UV-Vis equilibrium dialysis technique.

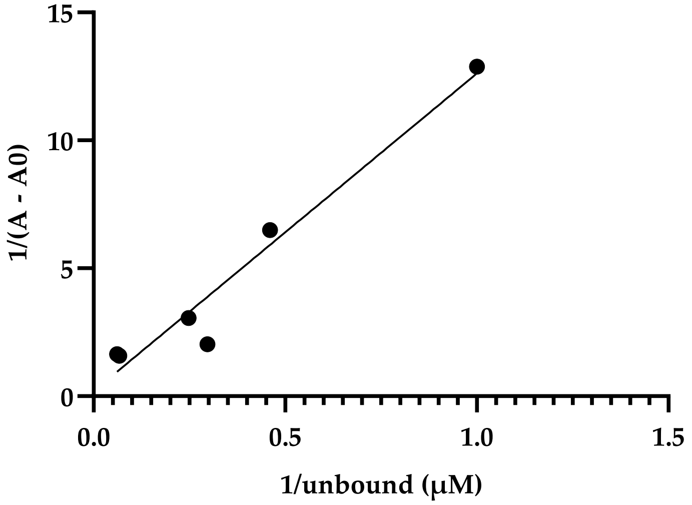

Figure S2. Double reciprocal plot of 1/(A-A0) versus 1/unbound.

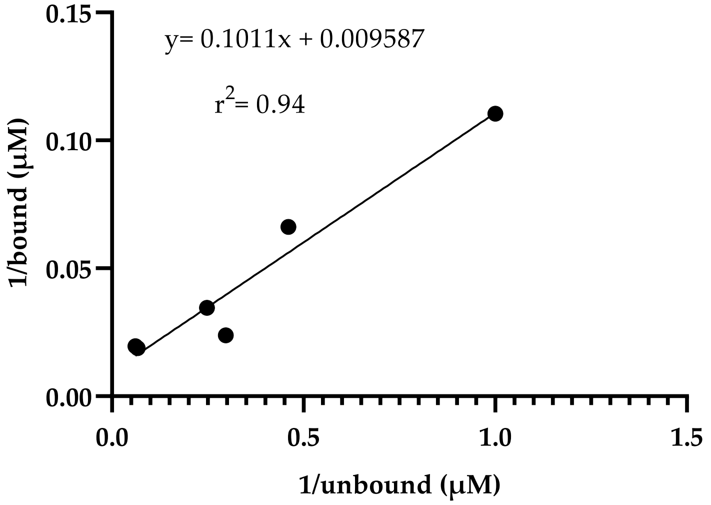

Figure S3. Double reciprocal plot of 1/bound ligand versus 1/unbound ligand.
